# Spatio-temporal trends of PCBs in the Swedish freshwater environment 1981–2012

**DOI:** 10.1007/s13280-014-0561-4

**Published:** 2014-11-15

**Authors:** Elisabeth Nyberg, Sara Danielsson, Ulla Eriksson, Suzanne Faxneld, Aroha Miller, Anders Bignert

**Affiliations:** 1 Department of Environmental Research and Monitoring, Swedish Museum of Natural History, Box 50007, 114 18 Stockholm, Sweden; 2 Department of Applied Environmental Science, Stockholm University, Svante Arrhenius väg 8, 106 91 Stockholm, Sweden; 3 Department of Applied Biology, University of British Columbia, 2357 Main Mall, Vancouver, BC V6T 1Z4 Canada

**Keywords:** Fish, PCBs, Monitoring, Freshwater, Sweden, Trends

## Abstract

Polychlorinated biphenyls (PCBs) have been monitored in perch (*Perca fluviatilis*), pike (*Esox lucius*), and Arctic char (*Salvelinus alpinus*) in reference lakes since the late 1960s. Temporal trends and spatial patterns are currently monitored in nine and 32 lakes, respectively. Overall, PCB concentrations are decreasing. However, this is not consistent for all congeners across all lakes and species. Perch has comparatively low PCB concentrations relative to suggested target levels, but individual congener concentrations in some lakes are concerningly high. No temporal trend is seen for CB-118 and CB-153 in perch, but significant decreasing trends exist for Arctic char and pike, for which monitoring started earlier than for perch. The lower/higher chlorinated congener ratio decreased over time in most lakes, indicating fewer new emissions. CB-118 and CB-153 concentrations in perch show spatial gradients across Sweden, with higher concentrations found near urban/industrial areas.

## Introduction

Polychlorinated biphenyls (PCBs) are one of the 12 groups of persistent organic pollutants (POPs) originally included in the Stockholm Convention on POPs.^1^ PCBs have been used in a wide variety of manufacturing processes, especially as plasticizers and insulators, and are widely distributed in the environment. In 1973, PCB use was banned in Sweden, except within sealed systems. In 1978, the ban was extended to prohibit all new use of PCBs.

PCBs can influence human health by affecting multiple organ systems (Carpenter 1998, 2006). Their toxicological effects on, for example, reproduction in mink are well documented (Aulerich and Ringer 1977; Bleavins et al. 1980). Animals in aquatic systems tend to biomagnify contaminants at a higher degree compared to terrestrial species due to the complexity and omnivory that characterizes aquatic food webs (Zanden and Rasmussen 1996). The concentrations of PCBs are generally positively correlated with the trophic position of a fish population within an aquatic food chain and are particularly high in predatory fish species (Brázová et al. 2012).

In the mid-1960s, research concerning environmental contaminant concentrations (e.g., chlorinated contaminants and heavy metals) and abundance, and their effects on wildlife began at the Swedish Museum of Natural History (SMNH) because of adverse health effects being observed in top predators such as Baltic grey seals (*Halichoerus grypus*) and white tailed sea eagles (*Haliaeetus albicilla*) (Helle et al. 1976a, b; Helander et al. 2002; Bredhult et al. 2008). During the 1970s, initial efforts were made to establish an environmental contaminant research program. In 1980, a comprehensive national monitoring program for environmental quality was formed by the Swedish Environmental Protection Agency (SEPA). SMNH was appointed the responsible institute for monitoring environmental contaminants in biological samples. The chemical analysis has been carried out at the same laboratory since the start of the monitoring program. The laboratory was initially a part of the SEPA and, since 1992, a part of Stockholm University (SU).

The primary objectives of the monitoring program were (1) to measure the concentrations of various contaminants and estimate normal variation in freshwater biota from representative sites throughout the country that were uninfluenced by local sources; (2) to describe the general contaminant load and to supply reference values that could be used for comparison with data from regional and local monitoring programs; (3) to monitor long-term time trends and estimate the rate of changes found; (4) to estimate the response in biota to actions taken to reduce the discharge of various contaminants; (5) to detect incidents of regional, national or international influence; (6) to detect renewed usage of banned contaminants for compliance monitoring, and (7) to discover large scale spatial differences across the country.

Sweden is a country with many lakes that cover more than 9 % of the country’s total surface area. The lakes within the Swedish National Monitoring Program for Contaminants in Freshwater Biota (SNMPCFB) are distributed from the northern parts of Sweden (Lake Abiskojaure) located 200 km north of the Arctic Circle, to the southern-most parts (Lake Krageholmssjön), with the majority located in the southern half of Sweden. The large distances between north and south leads to large temperature differences, thus some of the lakes are covered with ice for several months of the year, while others remain ice-free. The lakes also differ in size, nutrient status, general physical environment, and land use of surrounding areas. The smallest lake is 0.06 km^2^ (Lake Skärgölen) and the largest is 184 km^2^ (Lake Bolmen). These physical differences imply a great variability between the lakes concerning abiotic factors that might affect contaminant levels in fish.

Here we examine temporal and spatial relationships of PCB congeners (CB-28, CB-52, CB-101, CB-118, CB-138, CB-153 and CB-180) in pike (*E. lucius)*, Arctic char (*S. alpinus*) and perch (*P. fluviatilis*) to evaluate (1) concentrations over time in relation to imposed bans and restrictions; (2) spatial congener differences across Sweden; (3) concentrations against set environmental target levels; and (4) how monitoring design may affect the interpretation of these trends.

## Materials and methods

### Study species

The European perch is the most common freshwater fish in Sweden, and is also found in the brackish Baltic Sea (Kullander et al. 2012). Perch is an opportunistic predatory fish that undergoes an ontogenetic shift in diet (Collette et al. 1977; Kullander et al. 2012); small perch (5–30 mm) feed primarily on zooplankton, intermediate size (30–80 mm) on crustaceans, larvae and small fish, while large perch feed exclusively on fish and crayfish. Larger perch are thus exposed to biomagnifying substances at a high level of the aquatic food chain. The age at dietary shift is dependent on growth rate of perch, which can vary between lakes (Holmgren and Appelberg 2001). Perch muscle tissue is lean and contains approximately 0.3–0.6 % of extractable fat (Nyberg et al. 2013).

Pike is also a common fish in the Nordic countries and can be found in both fresh and brackish water (Baltic Sea). Pike is mainly piscivorous but occasionally also feeds on frogs, small mammals and sea birds (Kullander et al. 2012). As this species is located at a high trophic level, the concentrations of biomagnifying compounds such as POPs, are normally high. Pike is a lean fish with an average muscle fat content of 0.6 % (Nyberg et al. 2013).

Arctic char inhabits upland fresh waters of the Swedish mountain area. Diet varies depending on prey availability, fish size and the presence of other competitive species. Small individuals generally feed on benthic invertebrates and plankton, while larger individuals feed on fish, including conspecifics (Kullander et al. 2012). Because of the high trophic position of the large, piscivorous individuals, the concentrations of POPs might be high. Among the three investigated fish species, Arctic char has the highest lipid content: 1–3 % (Nyberg et al. 2013). All three species are fairly stationary, thus appropriate for studying local contaminant concentrations.

### Sampling sites, number of samples, and sampling frequency

**Fig. 1:**
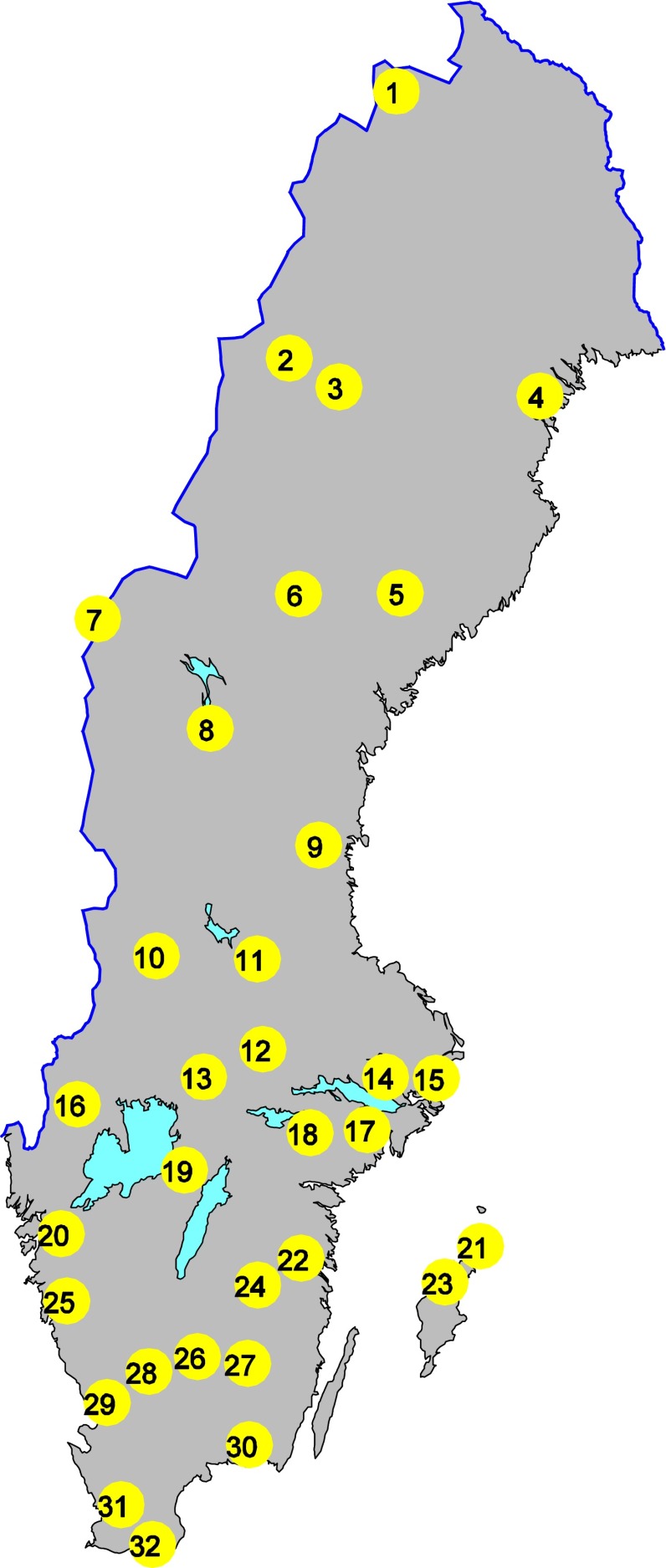
Sampling sites within the Swedish National Monitoring Program for Contaminants in Freshwater Biota. See Table 1 for information about the different lakes

A total of 32 lakes are included in the Swedish National Monitoring Program for Contaminants in Freshwater Biota (SNMPCFB). Approximately 20 of these lakes are located in the southern half of Sweden (Fig. 1). In general, only one species per lake was sampled, with three exceptions. The year of initial PCB analysis varied among the selected lakes (Table 1). To facilitate regional comparisons, selected lakes were chosen to avoid possible confounding factors that could influence contaminant concentration in the sampled fish tissues e.g., (1) lakes should not be influenced by local contamination and must have some protection against future exploitation, (2) land use surrounding the lakes should be well investigated and intensively farmed rural areas avoided, (3) areas of liming activities should be avoided, and (4) lakes should preferably be placed high in the drainage system and be oligo- or mesotrophic.

**Table 1:** Sampling sites, coordinates, species and number of years analyzed for PCBs (individual congeners) within the Swedish National Monitoring Program for Contaminants in Freshwater Biota. The first column refers to the sampling site numbers in Fig. 1

N in map	Lake	Latitude	Longitude	Species	Years analyzed for PCBs
1	Abiskojaure	68.31°	18.65°	Arctic char	81^a^, 86–98^a^, 00^a^, 07^a^, 08^b^, 09–10^c^, 11–12^d^
2	Tjulträsk	65.96°	16.07°	Arctic char	86–87^a^, 95^a^, 06^b^, 08–10^c^, 11–12^d^
3	Storvindeln	65.70°	17.13°	Pike	85–00^a^, 05^a^, 06^b^, 07^a^, 08^b^, 09^c^, 10^b^, 11–12^d^
4	Brännträsket	65.53°	21.42°	Perch	07^b^
5	Remmarsjön	63.86°	18.27°	Perch	00^a^, 05^a^, 07^b^
6	Degervattnet	63.87°	16.23°	Perch	05^a^, 07^b^
7	Stor-Björsjön	63.61°	12.23°	Arctic char	07^b^
8	Stor-Backsjön	62.68°	14.51°	Perch	07^b^
9	Stensjön	61.64°	16.58°	Perch	97–00^a^, 05^**a**^, 06^b^, 07^a^, 08^b^, 09^c^, 10^b^, 11–12^d^
10	Gipsjön	60.65°	13.63°	Perch	07^b^
11	Spjutsjön	60.64°	15.45°	Perch	07^b^
12	Övre Skärsjön	59.84°	15.55°	Perch	05^a^, 07^b^
13	Limmingsjön	59.59°	14.51°	Perch	07^b^
14	Fysingen	59.57°	17.92°	Perch	07^b^, 10^b^, 11–12^e^
15	Tärnan	59.56°	18.37°	Perch	05^a^, 07^b^
16	Bysjön	59.30°	12.34°	Perch	05^a^, 07^b^
17	Stora Envättern	59.11°	17.35°	Perch	00^a^, 05^a^, 07^b^
18	Älgsjön	59.09°	16.37°	Perch	07^b^
19	Svartsjön	58.76°	14.22°	Perch	07^b^, 11–12^d^
20	Fräcksjön	58.15°	12.18°	Perch	07^b^
21	Bästeträsk	57.92°	18.94°	Perch	07^b^
22	Allgjuttern	57.95°	16.10°	Perch	07^b^
23	Horsan	57.87°	18.84°	Perch	07^a^, 08^b^, 09^c^, 10^b^, 11–12^d^
24	Skärgölen	57.78°	15.58°	Perch	99^a^, 06^**b**^, 08^b^, 09–10^c^, 11–12^d^
25	Lilla Öresjön	57.56°	12.34°	Perch	07^b^
26	Fiolen	57.09°	14.53°	Perch	07^b^
27	Hjärtsjön	57.05°	15.26°	Perch	00^a^, 05^a^, 07^b^
28	Bolmen	56.97°	13.77°	Pike	88–00^a^, 05^a^, 06^b^, 07^a^, 08^b^, 09–10^c^, 11–12^d^
29	Stora Skärsjön	56.67°	13.07°	Perch	97–98^a^, 07^b^
30	Sännen	56.33°	15.36°	Perch	07^b^
31	Krankesjön	55.70°	13.47°	Perch	07^a^, 08^b^, 09^c^, 10^b^, 11–12^d^
32	Krageholmsjön	55.50°	13.75°	Perch	07^b^

^a^10 individual specimens per year, minor deviations occur

^b^1 pool per year of 10 individuals

^c^2 pools per year of 10 individuals per pool

^d^2 pools per year of 12 individuals per pool

^e^1 pool per year of 12 individuals

The earliest time-series for PCBs (ΣPCBs) in the freshwater environment are from the late 1960s (pike from two lakes, including one that represents the Arctic region in Sweden). The ΣPCBs were estimated from 14 peaks on a packed column GC after calibration with Aroclor 1254 (Jensen et al. 1983). During 1988, analysis on a capillary column was introduced, allowing analysis of individual congeners (Eriksson et al. 1994). Pike has been analyzed for PCBs on an individual congener basis at two sites, Lake Bolmen, since 1988, and Lake Storvindeln since 1985 (Fig. 1; Table 1). Pike was collected in spring (April–May), during or soon after spawning. Arctic char was analyzed for PCBs on an individual congener basis at three sites—Lake Abiskojaure since 1981 (analyzed retrospectively), Lake Tjulträsk since 1986 and Lake Stor-Björsjön since 2007 (Fig. 1; Table 1). Char was sampled in the autumn (August-November), usually during spawning. In recent years, perch was the most frequently sampled species and is currently collected from 27 lakes within the program (Fig. 1; Table 1). Perch has been analyzed for PCBs on an individual congener basis since 1997 in Lake Stensjön and 1999 in Lake Skärgölen (Table 1). Sampling of perch was carried out in autumn (August–October), outside of the spawning season.

Sampling has been carried out annually throughout the duration of the program. Prior to 2011, ten individuals of each species were analyzed annually from each lake, either individually or as a pooled sample. However, since 2011, twelve individuals have been analyzed as a pool (Table 1) (Bignert et al. 2013). A lower sampling frequency and sampling size than twelve individuals would result in a considerable decrease of statistical and interpretational power (Bignert et al. 1993). During 2001–2005, several of the collected samples were not analyzed but instead stored frozen at −20 or −80°C in the Environmental Specimen Bank (ESB). Samples from nine of the 32 lakes were analyzed annually for PCBs since 2007 (Lake Abiskojaure, Lake Bolmen, Lake Horsan, Lake Krankesjön, Lake Skärgölen, Lake Stensjön, Lake Tjulträsk, Lake Storvindeln, and Lake Svartsjön), with the exception of Lake Svartsjön, which was analyzed annually since 2011. Of these nine lakes, six have been analyzed for more than 10 years (Table 1). The rest of the 32 lakes have been analyzed for PCBs only one to three times since sampling started.

### Sample preparation and registered variables

For each fish, total body weight, body length, total length (body length plus the tail fin), sex, age, gonad weight, liver weight, and sample weight were recorded (Nyberg et al. 2013). To avoid surface contamination and to obtain a sample consisting of only muscle tissue, the epidermis and subcutaneous fatty tissue were carefully removed before the muscle tissue was excised. Muscle samples were taken from the middle dorsal muscle layer (TemaNord 1995). For the individual analyses, 10 g of muscle was taken from each fish; for the pooled samples, 1 g of muscle was taken from each fish (in total 10–12 g in each pool). The sampling and sample preparations were all performed according to the manual for collection, preparation, and storage of fish (SMNH 2012).

### Chemical analysis

Samples were extracted using a mixture of polar and non-polar solvents. The lipid content of the organic phase was determined gravimetrically. After clean-up of the dissolved lipid extracts using concentrated sulfuric acid, the samples were analyzed on a gas chromatograph equipped with a μ-electron capture detector and two 60 m columns with different polarity used in parallel (Jensen et al. 1983; Eriksson et al. 1997). One internal laboratory reference material (LRM) of muscle from fish was used at every extraction event since 1994. Four different materials have been used during this period with lipid content from 0.54 to 5.9 %. Within-laboratory reproducibility was calculated from the LRMs for more than 8000 PCB values for all analyzed congeners, and resulted in a reproducibility of 14 % for all reported PCB congener values between 2 and 50 ng g^−1^ lipid weight (l.w.) and 8 % for values above 50 ng g^−1^ l.w. The laboratory has participated in the periodic QUASIMEME (Quality Assurance of Information for Marine Environmental Monitoring in Europe) proficiency testing since 1993, with around 95 % of all reported values being within ±2 standard deviations of the assigned value. The quantification limit (defined as ten times the standard deviation of the measured concentration as the concentration approaches zero) is estimated to approximately 2 ng g^−1^ l.w. for all discussed PCB congeners.

### Statistical analysis and maps

For the temporal trend analysis, log-linear regression was performed for the entire investigated period and for the most recent 10 years using the yearly geometric mean values. In cases where the regression line had a poor fit, a 3-point running mean smoother was checked for statistical significance in comparison to the regression using ANOVA (Nicholson et al. 1998). Potential outliers in the temporal trends were detected as described in Hoaglin and Welsch (1978). Suspected outliers are indicated in the figures but were included in the statistical calculations. Values below level of quantification (LOQ) were replaced by LOQ divided by the square root of 2 prior to all statistical analyses. Power was fixed to 80 %. The minimum possible trend that could be detected during a 10-year monitoring period at a significance level of 5 % was estimated and power analysis was also carried out. A significance level of 5 % was used for all tests.

Spatial differences in PCB concentrations were evaluated using bar maps. The height of the bars represents the arithmetic mean for 2007–2012, or shorter if results were not available. Principal component analysis (PCA) was performed on the proportions of the individual PCB-congener concentrations to the ΣPCBs to study differences in the species congener patterns and differences due to latitude. The percentage of each PCB-congener relative to the sum of congeners was calculated and log-transformed prior to PCA analysis. Before the PCA-scores were plotted they were centered and scaled to 100 %. Hotelling’s *T* ^2^ test was used to check for possible significant differences in congener patterns. The statistical trend analysis and the bar maps were performed for the dl-PCB, CB-118 (2,3′,4,4′,5-pentachlorobiphenyl), and for the non dl-PCB CB-153 (2,2′,4,4′,5,5′-hexachlorobiphenyl), both of which are dominant congeners in fish, as well as the ratio between the more easily degradable CB-101 (2,2′4,5,5′-pentachlorobiphenyl) and the more stable CB-153. CB-28 (2,4,4′-trichlorobiphenyl) was included in the bar maps to show a divergent pattern. In the PCA analysis, CB-118 was chosen as the only dl-PCB, while CB-101, CB-153 and CB-180 (2,2′3,4,4′,5,5′-heptachlorobiphenyl) were chosen as representing PCBs with different degrees of chlorination. All statistics are based on lipid normalized values. Statistical software PIA (www.amap.no) was used for the trend analysis and the PCAs. All the results for perch (except within the PCA) are based on data from 2007 to 2012 because data are most complete during that period, while for Arctic char and pike, the results are based on data for the whole monitoring period.

### Environmental assessment criteria

In accordance with the Marine Strategy Framework Directive 2008/56/EC (MSFD), Good Environmental Status (GES) is defined as “concentrations of contaminants at levels not giving rise to pollution effects.” To determine GES, a number of target levels have been established representing a threshold that should not be exceeded. These target levels should protect the most sensitive organisms from the harmful effects of hazardous substances and have been developed within several groups or conventions e.g., Environmental Quality Standards (EQS) developed within the EC to evaluate GES (2008/105/EC), and the Environmental Assessment Criteria (EAC), developed within OSPAR (OSPAR 2009). For PCBs, EQSs are not established, so to evaluate concentrations of CB-118 and CB-153, OSPAR EACs are used in this study, and are 1.6 and 0.024 ug g^−1^ l.w., respectively (OSPAR 2009).

## Results

### Biological variables

Arithmetic mean weight, age, length and muscle fat content are presented for samples from all lakes within the SNMPFB (Table 2). Perch size was similar among the lakes because they are chosen to be of similar size. By contrast, the age difference was large (2.5–7.6 years) between the lakes, with perch from the north being older. This is expected because fish grow more slowly in colder climates.

**Table 2:** Arithmetic mean age, length, weight and extractable muscle fat content, ±95 % confidence intervals (CI) for samples within the Swedish National Monitoring Program in Freshwater Biota. For perch, the arithmetic mean value for 2007*–*2012 is presented. For Arctic char and pike, the arithmetic mean value for the whole monitoring period is presented. Presented in alphabetical order by lake

Lake	Species	Age (years) 95 % CI	Length (cm) 95 % CI	Weight (g) 95 % CI	Fat content (%) 95 % CI
Allgjuttern	Perch	4.6 (4.4–4.9)	18.0 (17.6–18.4)	56.6 (51.7–61.4)	0.54 (0.39–0.70)
Abiskojaure	Arctic char	5.1 (5.0–5.3)	26.9 (26.4–27.4)	216 (203–230)	1.7 (1.5–1.8)
Bolmen	Pike	5.2 (5.0–5.3)	54.1 (53.3–54.9)	1080 (1040–1120)	0.55 (0.54–0.57)
Brännträsket	Perch	7.6 (7.2–8.1)	18.7 (18.3–19.1)	68.1 (63.3–72.8)	0.62 (0.28–0.96)
Bysjön	Perch	5.4 (5.1–5.7)	17.3 (17.0–17.6)	52.9 (49.8–56.0)	0.68 (0.36–0.99)
Bästeträsk	Perch	3.9 (3.7–4.1)	17.5 (17.3–17.8)	53.4 (51.2–55.6)	0.70 (0.37–1.0)
Degervattnet	Perch	5.5 (5.2–5.8)	18.3 (18.0–18.6)	63.8 (60.6–67.0)	0.48 (0.31–0.65)
Fiolen	Perch	4.4 (4.2–4.7)	18.0 (17.6–18.3)	61.5 (57.5–65.5)	0.60 (0.43–0.76)
Fräcksjön	Perch	5.3 (5.0–5.5)	17.1 (16.8–17.4)	51.9 (48.3–55.5)	0.58 (0.43–0.76)
Fysingen	Perch	4.7 (4.4–4.9)	17.1 (16.8–17.4)	53.4 (50.0–56.8)	0.54 (0.46–0.62)
Gipsjön	Perch	6.4 (6.2–6.6)	17.3 (17.0–17.7)	56.1 (52.7–59.5)	0.52 (0.41–0.62)
Hjärtsjön	Perch	3.6 (3.4–3.7)	18.7 (18.3–19.0)	68.7 (64.9–72.6)	0.76 (0.44–1.1)
Horsan	Perch	5.3 (5.1–5.6)	19.0 (17.6–20.4)	58.4 (55.6–61.3)	0.68 (0.62–0.74)
Krageholmsjön	Perch	2.5 (2.3–2.7)	17.5 (16.9–18.1)	70.6 (61.0–80.2)	0.63 (0.49–0.77)
Krankesjön	Perch	3.1 (3.0–3.3)	19.5 (16.5–22.5)	59.6 (56.6–62.6)	0.50 (0.45–0.56)
Lilla Öresjön	Perch	5.6 (5.3–5.9)	18.3 (18.0–18.7)	63.5 (59.2–67.8)	0.70 (0.37–1.02)
Limmingsjön	Perch	4.9 (4.7–5.2)	17.5 (17.2–17.9)	55.8 (52.7–58.9)	0.65 (0.54–0.76)
Remmarsjön	Perch	6.8 (6.5–7.2)	18.9 (18.4–19.4)	72.7 (67.3–78.0)	0.66 (0.53–0.78)
Skärgölen	Perch	4.7 (4.5–5.0)	17.8 (16.9–18.7)	59.0 (54.8–63.1)	0.68 (0.64–0.72)
Spjutsjön	Perch	3.9 (3.7–4.1)	18.2 (17.9–18.4)	62.0 (59.1–64.9)	0.72 (0.52–0.92)
Stora Envättern	Perch	5.3 (5.1–5.6)	16.8 (16.5–17.2)	47.8 (44.2–51.4)	0.62 (0.30–0.93)
Stensjön	Perch	7.4 (7.0–7.7)	19.0 (18.3–19.7)	63.3 (61.1–65.6)	0.60 (0.54–0.65)
Stora Skärsjön	Perch	6.3 (6.1–6.6)	16.4 (16.2–16.6)	46.3 (44.4–48.2)	0.56 (0.45–0.68)
Stor–Backsjön	Perch	6.5 (6.2–6.7)	18.4 (18.1–18.6)	63.8 (61.1–66.5)	0.65 (0.47–0.82)
Stor–Björsjön	Arctic char	5.6 (4.5–6.7)	26.3 (25.6–27.1)	160 (146–175)	1.0 (0.46–1.6)
Storvindeln	Pike	5.8 (5.6–5.9)	61.3 (60.7–62.0)	1630 (1570–1680)	0.58 (0.57–0.60)
Svartsjön	Perch	7.3 (6.5–8.1)	15.6 (14.7–16.5)	39.1 (31.0–47.1)	0.70 (0.59–0.80)
Sännen	Perch	5.7 (5.5–6.0)	17.0 (16.7–17.3)	47.5 (44.1–51.0)	0.59 (0.39–0.79)
Tjulträsk	Arctic char	5.2 (5.1–5.3)	26.9 (26.3–27.6)	209 (193–225)	1.5 (1.3–1.6)
Tärnan	Perch	5.9 (5.6–6.2)	17.7 (17.3–18.1)	58.1 (53.1–63.0)	0.60 (0.50–0.69)
Älgsjön	Perch	5.8 (5.4–6.1)	16.7 (16.3–17.0)	46.1 (43.4–48.8)	0.69 (0.47–0.90)
Övre Skärsjön	Perch	6.4 (6.1–6.7)	18.5 (18.2–18.8)	64.6 (61.1–68.1)	0.63 (0.58–0.67)

For Arctic char, both the age and total length were very similar between the three sampled lakes. For pike, specimens from Lake Storvindeln were somewhat larger and less lean than pike from Lake Bolmen.

### Temporal trends

**Fig. 2:**
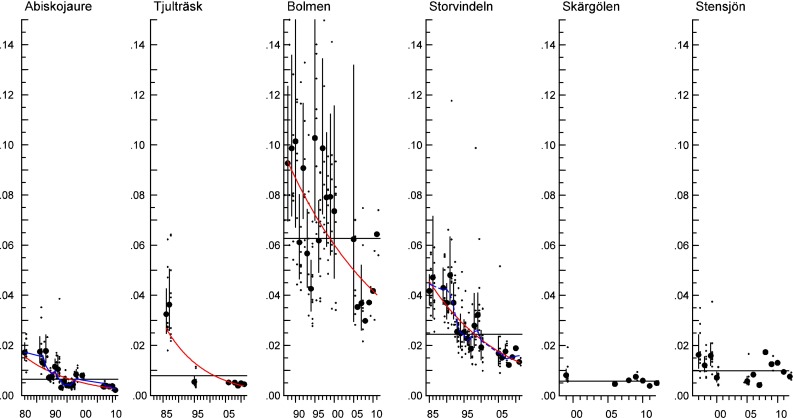
Log-linear trends of CB-118 (ug g^−1^ lipid weight) in Arctic char muscle from Lake Abiskojaure and Lake Tjulträsk; in pike muscle from Lake Bolmen and Lake Storvindeln; and in perch muscle from Lake Skärgölen and Lake Stensjön (time series starting in 1981, 1986, 1988, 1985, 1997 and 1999, respectively). The *red lines* show a significant trend over the whole period and for the ten last years. The *dark blue lines* indicate non-linear trends (0.05 < *p* < 0.1). The *black horizontal line* shows the mean concentration over the whole period. Each figure displays the geometric mean concentration of each year (*circles*) together with the individual analyses (*small dots*) and the 95 % confidence intervals of the geometric means

**Fig. 3:**
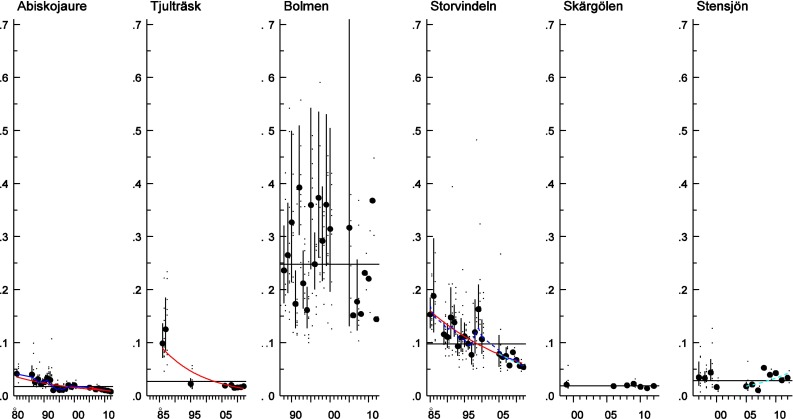
Log-linear trends of CB-153 (ug g^−1^ lipid weight) in Arctic char muscle from Lake Abiskojaure and Lake Tjulträsk; in pike muscle from Lake Bolmen and Lake Storvindeln; and in perch muscle from Lake Skärgölen and Lake Stensjön (time series starting in 1981, 1986, 1988, 1985, 1997 and 1999, respectively). The *red lines* show significant linear trends over the whole period. The *light blue dotted line* indicates a trend for the last ten years (0.05 < *p* < 0.2). The *dark blue lines* indicate non-linear trends (0.05 < *p* < 0.1) and the *black horizontal line* the mean concentration over the whole period. Each figure displays the geometric mean concentration of each year (*circles*) together with the individual analyses (*small dots*) and the 95 % confidence intervals of the geometric means

In perch muscle samples, no trends were seen for dl CB-118 in either sampled lake, but significant decreasing trends were seen for both Arctic char and pike muscle samples, of between 4.2 and 7.8 % per year (Fig. 2; Table 3). As with CB-118, no trends were seen for CB-153 in perch muscle from Lake Skärgölen or Lake Stensjön over the whole period (Fig. 3), but the statistical power to detect trends was relatively low. Concentrations of CB-153 in Arctic char decreased significantly in both lakes (between 4.4 and 7.2 % per year). CB-153 in pike showed no trend in Lake Bolmen, but decreased significantly in Lake Storvindeln (3.6 % per year) (Fig. 3; Table 3).

**Table 3:** Lake, species, contaminant, the annual percentage change ±95 % confidence interval (CI), *r* ^2^ reporting the coefficient of determination, *p* value (significant at <0.05), the lowest detectable change (% per year) for a ten year period with the current between year variation at a power of 80 %, coefficient of variation (CV) around the regression line as a measure of between-year variation, and the number of years required to detect a trend of 10 % at a power of 80 % for CB-118, CB-153 and the ratio between CB-101:CB-153 in Arctic char, pike and perch (muscle). Data for the whole monitoring period are presented in the first row and for the most recent 10 years (2003–2012) in the lower row for each lake. Presented in order of contaminant

Lake	Species	Contaminant	Annual % change (95 % CI)	*r* ^2^	*p* value	Lowest detectable change (%)	CV (%)	*N* years required to detect a trend of 10 %
Abiskojaure	Arctic char	CB-118						
1981–2012			−5.8 (−7.8, −3.9)	0.67	<0.001	14	40	13
2003–2012			−10 (−22, 2.2)	0.57	0.084	6.7	19	9
Tjulträsk	Arctic char	CB-118						
1986–2012			−7.8 (−11, −4.7)	0.83	<0.001	15	40	13
2003–2012			−5.0 (−12, 1.5)	0.54	0.098	4	11	7
Bolmen	Pike	CB-118						
1988–2012			−4.2 (−6.0, −2.4)	0.55	<0.001	12	32	11
2003–2012			−4.3 (−18, 9.5)	0.08	0.48	14	38	12
Storvindeln	Pike	CB-118						
1985–012			−4.9 (−6.0, −3.9)	0.83	<0.001	6.9	19	9
2003–2012			−4.4 (−12, 2.7)	0.28	0.18	6.9	19	9
Skärgölen	Perch	CB-118						
1999–2012			−3.6 (−9.1, 2.0)	0.35	0.16	8.4	24	10
2003–2012			−2.0 (−17, 13)	0.03	0.72	9.4	26	10
Stensjön	Perch	CB-118						
1997–2012			−2.1 (−7.8, 3.6)	0.06	0.44	17	48	14
2003–2012			6.7 (−11, 24)	0.13	0.39	18	49	14
Abiskojaure	Arctic char	CB-153						
1981–2012			−4.4 (−5.9, −2.9)	0.64	<0.001	11	31	11
2003–2012			−8.7 (−13, −4.8)	0.87	<0.01	3.2	9.0	6
Tjulträsk	Arctic char	CB-153						
1986–2012			−7.2 (−10, −4.5)	0.85	<0.001	13	36	12
2003–2012			−2.5 (−9.4, 4.6)	0.20	0.38	4.4	13	7
Bolmen	Pike	CB-153						
1988–2012			−1.2 (−3.2, 0.74)	0.08	0.33	12	34	12
2003–2012			−0.1 (−14, 14)	0.00	0.95	14	39	13
Storvindeln	Pike	CB-153						
1985–012			−3.6 (−4.6, −2.6)	0.73	<0.001	6.9	19	9
2003–2012			−4.7 (−9.5, 0.18)	0.48	0.055	4.6	13	7
Skärgölen	Perch	CB-153						
1999–2012			−1.6 (−5.2, 2.0)	0.21	0.30	5.4	15	8
2003–2012			−2.6 (−12, 6.9)	0.13	0.49	5.9	17	8
Stensjön	Perch	CB-153						
1997–2012			0.57 (−5.9, 7.0)	0.00	0.83	19	55	15
2003–2012			12 (−6.3, 31)	0.30	0.16	19	52	25
Abiskojaure	Arctic char	CB-101/CB-153						
1981–2012			−2.4 (−3.4, −1.5)	0.59	<0.001	6.7	19	9
2003–2012			2.6 (−4.6, 9.8)	0.15	0.40	6.0	17	8
Tjulträsk	Arctic char	CB-101/CB-153						
1986–2012			−0.98 (−2.0, 0.052)	0.42	0.058	4.6	13	7
2003–2012			0.37 (−5.9, 6.7)	0.00	0.85	3.9	11	7
Bolmen	Pike	CB-101/CB-153						
1988–2012			−2.5 (−3.2, −1.7)	0.71	<0.001	4.6	13	7
2003–2012			−2.7 (−8.4, −2.9)	0.19	0.28	5.4	15	8
Storvindeln	Pike	CB-101/CB-153						
1985–012			−2.0 (−2.7, 1.3)	0.63	<0.001	4.8	13	7
2003–2012			−0.41 (−2.2, 1.4)	0.04	0.61	1.7	4.9	5
Skärgölen	Perch	CB-101/CB-153						
1999–2012			−1.9 (−5.4, 1.5)	0.29	0.21	5.1	14	7
2003–2012			0.60 (−7.8, 9.0)	0.01	0.83	5.2	15	8
Stensjön	Perch	CB-101/CB-153						
1997–2012			−2.5 (−4.1, −0.97)	0.57	<0.01	4.4	12	7
2003–2012			−4.9 (−9.4, −0.34)	0.54	<0.05	4.3	12	7

The number of years to detect a significant annual change of 10 % with 80 % statistical power for CB-153 and CB-118 varied: 11–13 years in Arctic char, 9–12 years in pike and 8–15 years in perch (Table 3). The statistical power to detect an annual change of 10 % was very close to 100 % in both the pike and Arctic char time series from Lake Abiskojaure for the entire period (not presented). Statistical power for the shorter perch time series and the Arctic char series from Lake Tjulträsk, which have fewer data points, varied between 37 and 77 %.

**Fig. 4:**
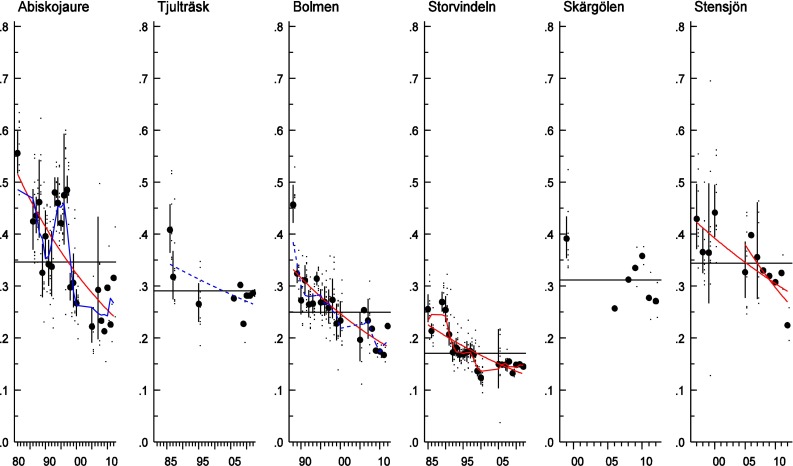
Log-linear trends of the CB-101/CB-153-ratio in Arctic char muscle from Lake Abiskojaure and Lake Tjulträsk; in pike muscle from Lake Bolmen and Lake Storvindeln; and in perch muscle from Lake Skärgölen and Lake Stensjön (time series starting in 1981, 1986, 1988, 1985, 1997 and 1999, respectively). The *red linear and non-linear lines* show a significant trend over the whole period and for the ten last years. The *dark blue lines* indicate non-linear trends (0.05 < *p* < 0.1). The *black horizontal line* shows the mean concentration over the whole period. Each figure displays the geometric mean concentration of each year (*circles*) together with the individual analyses (*small dots*) and the 95 % confidence intervals of the geometric means

The ratio between the penta- and hexa-PCBs, as illustrated by CB-101/CB-153 (Fig. 4) has decreased over time in most lakes (between 2.0 and 2.5 % per year), with the exception of Lake Skärgölen and Lake Tjulträsk, where there are few data points.

### Spatial patterns

**Fig. 5:**
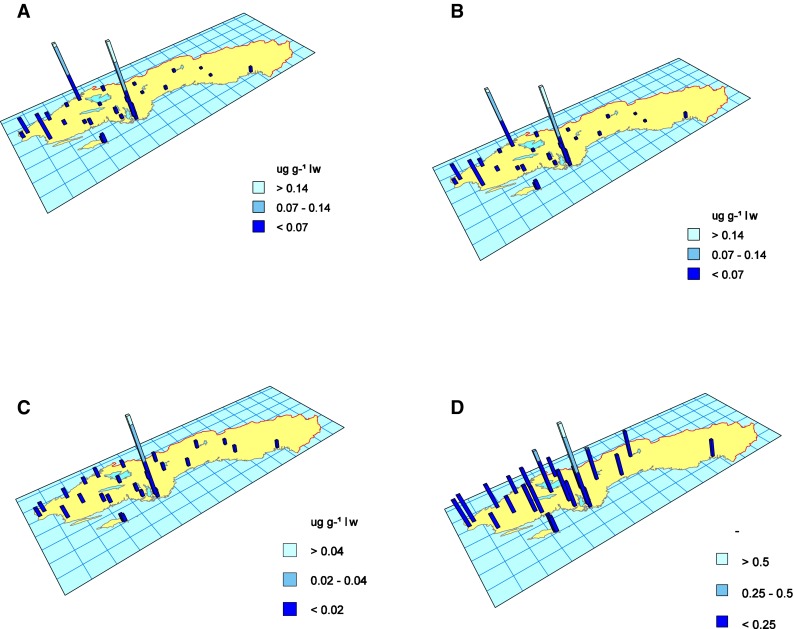
Spatial variation in concentration (ug g^−1^ lipid weight) in freshwater perch muscle of **a** CB-153 **b** CB-118, **c** CB-28 and **d** the ratio between CB-101/CB-153; arithmetic mean values from 2007 to 2012

For both CB-153 and CB-118 (Fig. 5a, b) concentrations in perch muscle were lowest in lakes in the north of Sweden, highest around urban centres of the three largest cities in Sweden, Stockholm, Gothenburg and Malmö, as well as in Karlskrona, an old naval city. Congener pattern differed between lakes. Lake Fysingen stands out with relatively high concentrations of the low chlorinated CB-28 (Fig. 5c). A similar pattern was observed when looking at the spatial distribution of CB-101/CB-153. Lower ratios were observed in the rural areas and higher ratios were observed in more densely populated regions (Fig. 5d).

**Fig. 6:**
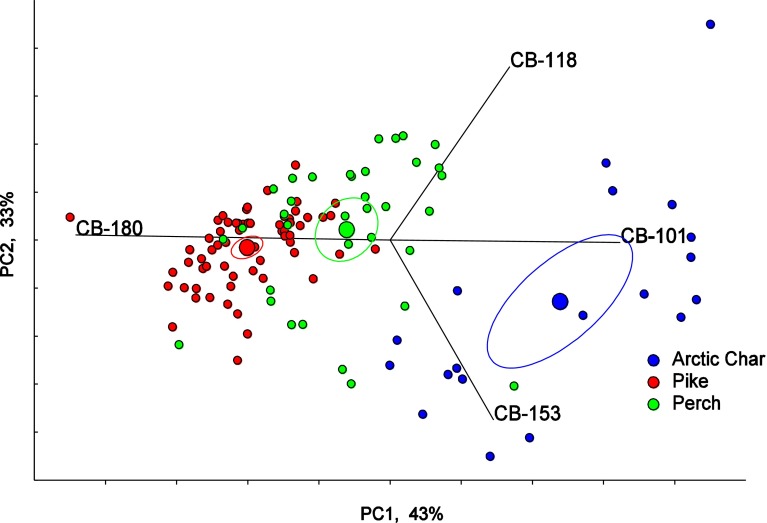
PCA (principal component analysis), biplot and Hotellings 95 % confidence ellipses for center of gravity for each group. The figure shows PCB congeners (CB-101, CB-118, CB-153 and CB-180) in Arctic char, pike and perch (1997–2012) from the northern parts of Sweden, north between 61.00° and 69.00° latitude

Clear differences in congener pattern were seen between Arctic char and pike from the northern regions of Sweden (Fig. 6a). Pike generally has higher relative concentrations of CB-180 compared to the other two species, while Arctic char has relatively higher concentrations of CB-101. Perch from northern Sweden had a congener pattern between pike and Arctic char, and was significantly different from both of those species (*p* < 0.05, Hotelling’s *T* ^2^ test).

### Target levels

In all lakes and species, CB-153 concentration is below the OSPAR EAC of 1.6 ug g^−1^ l.w. (Figs. 3, 5a). The EAC for CB-118 of 0.024 ug g^−1^ lipid weight was exceeded in pike from Lake Bolmen and perch from Lake Krankesjön, Lake Sännen, Lake Fräcksjön, Lake Tärnan and Lake Fysingen (Figs. 2 and 5b) in 2007–2012.

## Discussion

The concentrations of PCBs, illustrated here by CB-118 and CB-153, were generally decreasing by about 3–8 % per year in both pike and Arctic char. No trend was observed for the perch time series, but this is most likely due to the short duration of these time series and because monitoring in perch started after the steep decrease, during the 1980s and 1990s, observed for pike and Arctic char. Decreasing trends for PCBs in biological samples of similar magnitudes have been reported in studies from the Baltic Sea (Bignert et al. 2013; Miller et al. 2013) and other countries (e.g., Braune et al. 2005; Ryan et al. 2005; Helgason et al. 2008; Rigét et al. 2010). In a review on temporal trends of PCBs in arctic biota, Rigét et al. (2010) found a mean annual decrease for CB-153 of 1.2 % based on all the time series analyzed in the review (40 in total), which was somewhat lower than the decrease found in the time series here. A number of the time series presented in Rigét et al. (2010) started in the 1990s, later than the time series of our study, the concentrations were lower and the decrease less steep, which might explain the different results and the mean power in our time series were also considerably higher, 71 % compared to 28 % in Rigét et al. (2010).

The decrease over time, seen for concentrations of PCBs in both freshwater and marine fish in Sweden, mirrors the measures taken (e.g., bans and restrictions) to reduce PCBs in the environment. Gewurtz et al. (2010) found similar results as in our study for ∑PCB, a steep decrease in the 1970s and 1980s, which levelled out in the mid-1990s, most probably as a result of bans and restrictions.

The ratio between the penta- (CB-101) and hexa-PCBs (CB-153) has decreased over time in most lakes. This decrease was expected due to a higher degree of volatilization and degradation of lower chlorinated PCBs. A decreasing ratio indicates that a temporal removal from the source has occurred. When locally contaminated lakes are identified, an increase in this ratio can be observed. During the last decade, the decrease appears to have levelled out at Lake Storvindeln and Lake Abiskojaure, which might indicate some change in PCB source in these areas. It is important that further studies are conducted to monitor the ratio of higher and lower chlorinated PCBs, as this may indicate releases of PCBs from new sources.

The concentrations of most PCBs (shown by CB-153 and CB-118) differ among lakes. It appears that lakes in the vicinity of urban and/or industrial areas (e.g., Lake Fysingen close to Arlanda airport and Lake Sännen close to the old naval city of Karlskrona), appear to be at greater risk of higher concentrations of PCBs compared to lakes in rural, less densely populated regions. Lake Fysingen stands out with not just high concentrations of CB-153 and CB-118, but also a high ratio of CB-101/CB-153 and relatively high concentrations of the lower chlorinated CB-28, indicating a new input of PCB. This needs to be investigated further to find out if this implies exposure from a new source or changes in land use around the lake. Turrio-Baldassarri et al. (1997) found that a relation between a persistent and a less persistent CB-congener could indicate new contamination using the ratio between CB-149/CB-153 in cow milk.

The principal component analysis on perch, pike, and Arctic char from the northern parts of Sweden showed clear differences in congener pattern between Arctic char and pike, which might be explained by differences in diet, uptake of contaminants and metabolism. Perch also differed from both pike and Arctic char, but were more similar to pike. Babut et al. (2012) found that fish ecological traits are important factors that could explain differences in the bioaccumulation of PCBs when examining 2848 samples of 36 freshwater fish species from approximately 300 sites in France. However, Babut et al. (2012) also found that congener pattern in different fish species was more related to their physiology and metabolism than to their ecological traits. Arctic char in our study showed a lot of within-species variance, which could be expected because this species varies to a great extent both between and within lakes, likely because these species are present in more lake habitats and feed at more variable trophic levels compared with the other two species (Kullander et al. 2012).

The EAC for CB-118 was exceeded in pike from Lake Bolmen and perch from Lake Krankesjön, Sännen, Fräcksjön, Tärnan and Fysingen, which shows that the levels were still too high in some parts of the freshwater environment to protect the most sensitive organisms. Fish from Latvian lakes (including perch) show similar levels to that observed here for CB-118, with a mean value of 0.026 ug g^−1^ l.w. (recalculated from fresh weight to lipid weight basis) in fish muscle, which also exceeds the EAC (Zacs et al. 2013).

After >40 years of freshwater monitoring, some valuable lessons have been learned for developing the monitoring design of POPs. These include:

- Examination of individual congeners rather than only looking at the summed concentrations of a substance.
- The importance of annual monitoring.
- The choice of monitoring species, organ and selection of samples concerning e.g., age, sex, size, and sampling season.
- The importance of using the same laboratory for contaminant monitoring for temporal trend studies.

With a well-designed monitoring programme, small changes over time and space can be detected faster and this allows for focused remediation and policy efforts in specifically identified areas.

## Footnotes

1
http://chm.pops.int/TheConvention/ThePOPs/ListingofPOPs/tabid/2509/Default.aspx.
